# 2811. Real-World Experience Utilizing Cefiderocol for Serious Multidrug Resistant Gram-Negative Infections

**DOI:** 10.1093/ofid/ofad500.2422

**Published:** 2023-11-27

**Authors:** Nicholas S Teran, Andrei Zidaru, Kady Phe

**Affiliations:** Baylor St. Luke's Medical Center & University of Houston, Houston, Texas; Baylor St. Luke's Medical Center, Houston, Texas; Baylor St. Luke's Medical Center, Houston, Texas

## Abstract

**Background:**

Cefiderocol is a novel siderophore cephalosporin that demonstrated in vitro activity against carbapenem-resistant Enterobacterales, *Acinetobacter baumannii* (AB), *Pseudomonas aeruginosa* (PA), and *Stenotrophomonas maltophilia.* Cefiderocol demonstrated promising efficacy in randomized, clinical trials; however, real-world utilization of cefiderocol is not well characterized.

**Methods:**

This was a retrospective study of adults who received cefiderocol for ≥72h for serious meropenem-resistant Gram negative infections at our institution between 11/2021 and 12/2022. Patients with all sources of infection were considered for inclusion. Clinical failure was defined as all-cause 90d or in-hospital mortality, or the lack of resolution of signs or symptoms of infection. Microbiological failure was defined as positive cultures growing the index pathogen after ≥7d of initiating therapy or ≤60d of completing therapy. Descriptive statistics were used to compare outcomes by the index pathogen.

**Results:**

Of 55 patients who received cefiderocol, 35 patients were included. The remaining 20 patients were excluded for receiving < 72h of therapy. The most common infections were pneumonia (n = 17, 48.6%), skin and skin structure infections (n = 6, 17.1%), and osteomyelitis (n = 5, 14.3%) wherein PA (n = 17, 48.6%) and AB (n = 14, 40%) were isolated most frequently. The overall 90d and in-hospital mortality rates were 9 (25.7%) and 11 (31.4%) patients, respectively. Clinical failure, assessed in 34 patients, occurred in 10 (29.4%) patients and was similar between pathogens (p = 0.71). Microbiological failure, assessed in 26 patients, occurred more commonly in infections caused by PA (n = 11/12, 91.7%) compared to all other pathogens (n = 3/14, 21.4%; p < 0.01). Notably, most microbiological failures due to PA were in the setting of pneumonia (n = 6/11, 54.5%).

**Conclusion:**

Real-world experience demonstrated that most patients receiving cefiderocol for serious Gram-negative infections did well; however, microbiological failure among patients with PA infections may be attributed to the difficulty in differentiating true pathogens and colonizers in respiratory infections.

**Disclosures:**

**All Authors**: No reported disclosures

